# Development of a human-size magnetic particle imaging device for sentinel lymph node biopsy of breast cancer

**DOI:** 10.3389/fbioe.2024.1327521

**Published:** 2024-02-13

**Authors:** Shi Bai, Lingke Gai, Qinyang Zhang, Yue Kang, Zhiyao Liu, Yuan He, Wenzhong Liu, Tao Jiang, Zhongzhou Du, Siyao Du, Si Gao, Min Zhang, Tianshu Li

**Affiliations:** ^1^ School of Information Science and Engineering, Shenyang University of Technology, Shenyang, China; ^2^ Liaoning Magnetic Medical Sensing and Treatment Technological Innovation Center, Liaoning Jiayin Medical Technology Co., Ltd., Shenyang, China; ^3^ Department of Breast Surgery, Cancer Hospital of Dalian University of Technology, Cancer Hospital of China Medical University, Liaoning Cancer Hospital & Institute, Shenyang, China; ^4^ Faculty of Science and Technology, Keio University, Yokohama, Japan; ^5^ School of Artificial Intelligence and Automation, Huazhong University of Science and Technology, Wuhan, China; ^6^ School of Biomedical Engineering and Informatics, Nanjing Medical University, Nanjing, China; ^7^ Department of Computer and Communication Engineering, Zhengzhou University of Light Industry, Zhengzhou, China; ^8^ First Affiliated Hospital, China Medical University, Shenyang, China

**Keywords:** human-sized MPI, field free point, third-harmonic signal, SPIONs, sentinel lymph node biopsy

## Abstract

In this study, a novel human-size handheld magnetic particle imaging (MPI) system was developed for the high-precision detection of sentinel lymph nodes for breast cancer. The system consisted of a highly sensitive home-made MPI detection probe, a set of concentric coils pair for spatialization, a solenoid coil for uniform excitation at 8 kHz@1.5 mT, and a full mirrored coil set positioned far away from the scanning area. The mirrored coils formed an extremely effective differential pickup structure which suppressed the system noise as high as 100 dB. The different combination of the inner and outer gradient current made the field free point (FFP) move in the Z direction with a uniform intensity of 0.54T/m, while the scanning in the XY direction was implemented mechanically. The third-harmonic signal of the Superparamagnetic Iron Oxide Nanoparticles (SPIONs) at the FFP was detected and then reconstructed synchronously with the current changes. Experiment results showed that the tomographic detection limit was 30 mm in the Z direction, and the sensitivity was about 10 μg Fe SPIONs at 40 mm distance with a spatial resolution of about 5 mm. In the rat experiment, 54 μg intramuscular injected SPIONs were detected successfully in the sentinel lymph node, in which the tracer content was about 1.2% total injected Fe. Additionally, the effective detection time window was confirmed from 4 to 6 min after injection. Relevant clinical ethics are already in the application process. Large mammalian SLNB MPI experiments and 3D preoperative SLNB imaging will be performed in the future.

## 1 Introduction

Sentinel lymph node biopsy (SLNB) is a standard diagnostic procedure in breast cancer surgery, used to determine the staging of breast cancer and the extent of surgical resection. Traditional dyeing methods such as methylene blue and nano-carbon require careful observation during the surgery; therefore, it prolongs surgery time significantly and the SLNB accuracy relies on physician experience heavily. Moreover, another SLNB technology named the Tc-99m nuclide method cannot be popularized in most regions due to use regulations (Krag et al., 1993; Temple et al., 2002; Wydra et al., 2005; Swenson et al., 2002; Madsen et al., 2008). In recent years, a new SLNB technology using superparamagnetic iron oxide nanoparticles (SPIONs) as tracers was proposed, which has expected widespread attention because its safety and accuracy are superior to traditional methods (Shiozawa et al., 2013). A SPIONs SLNB device named Sentimag^®^ was developed, as well as Sienna+^®^, a paired magnetic tracer, which has been studied in the multicenter Sentimag trial (Douek et al., 2014). However, the fundamental harmonic-based signal acquisition method and non-differential sensor structure make them extremely susceptible to external interference, such as surgical forceps or power disturbances, and they do not have the ability to perform tomographic scanning.

Another handheld magnetic probe based on static magnetic induction technology has also been developed to identify sentinel lymph nodes. However, the static sensing method limited the detection sensitivity, depth, and tomographic ability of magnetic SLNB. The maximum detection performance was about 10 mm depth for 140 μg Fe which is not enough for preoperative detection (Sekino et al., 2018).

In this study, a novel magnetic SLNB developed for human SLNB application based on magnetic particle imaging (MPI) technology was presented. MPI is a new tomographic SPIONs imaging method that was first proposed in 2005 (Gleich and Weizenecker, 2005). Through 20 years of rapid development, MPI has become an attractive new imaging technology worldwide (Sattel et al., 2008; Knopp and Buzug, 2012; Tonyushkin, 2017; Yu et al., 2017; Wang et al., 2020; Chandrasekharan et al., 2020). Bruker and UC Berkeley have launched their preclinical MPI products in recent years with a closed-bore field of view (FOV). However, there are still several important problems for most of the current MPI research, namely, the limited closed-bore FOV as well as the overlarge excitation AC field that exceeds the human safety threshold in order to expand the FOV. Therefore, MPI has not entered into practical clinical application so far.

Different to traditional closed-bore MPI, we developed a single-sided MPI system in this study for human SLNB application. The developed open-bore single-sided structure was very suitable for local tissue imaging as well as the excitation safety. *In vitro* and *in vivo* rat experiments indicated that the developed MPI SLNB system provided superior performance compared to current magnetic SLNB devices, and the follow-up clinical research preparations are also in progress.

## 2 Materials and system development

### 2.1 SPIONs materials

A superparamagnetic nanoparticle named Resovist (Fujifilm, Japan), which was originally developed as an MRI T2 contrast agent, was used as tracer in the present study. Resovist was selected here for its safety, excellent harmonic magnetic response, and its hydrodynamic diameter, which is most important in SLNB diagnosis. As known, the gap of a capillary wall is less than 50 nm, while it is much larger for lymphatic vessels. Generally, a nanoparticle between 50 and 150 nm can penetrate the lymphatic wall smoothly but be blocked by the capillary wall. The average hydrodynamic diameter of the Resovist is about 57 nm (Dynamic light scattering (DLS) measurement), thus it is a very suitable tracer for MPI SLNB applications. The elimination of Resovist is mainly through the hepatobiliary system (liver and bile). It is excreted into the bile, and from there, it eventually leaves the body through feces. The maximum iron dose administered per Resovist injection is 44.6 mg, akin to the daily iron intake for the human body. This dosage exerts no discernible impact on the coloration of the patient’s urine, or, at most, induces only minimal color variations. The minimum and maximum effective times for clinical SLNB localization using Resovist as a tracer were 5 min and 24 h in previous studies (Taruno et al., 2019).

### 2.2 Human-sized SLNB MPI system

**FIGURE 1:**
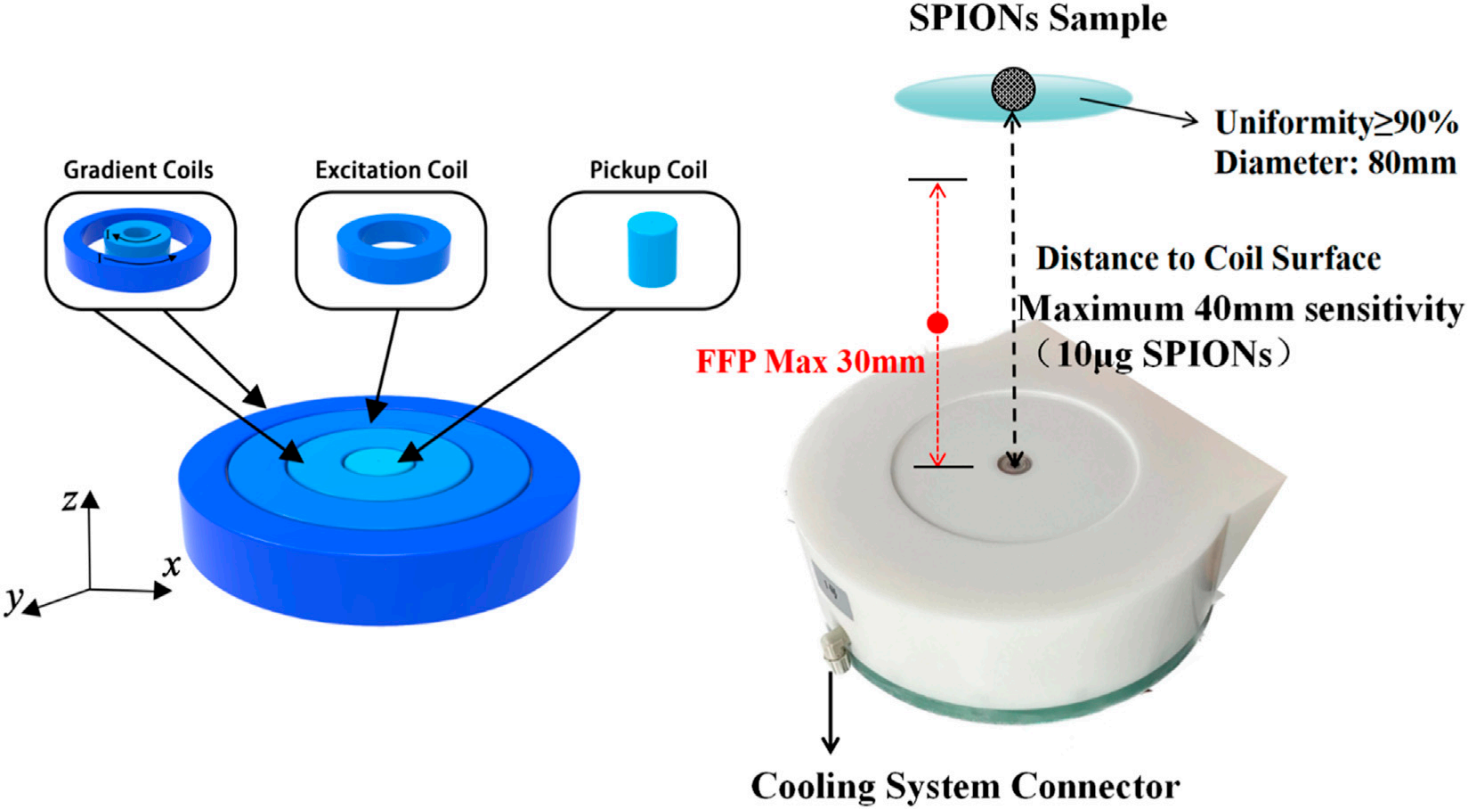
Schematic diagram and photograph of the excitation, gradient, and pickup coils without differential structures. The FFP with a uniform strength was obtained from 0 to 30 mm in the Z direction along the central axis. All coils were packaged in a high strength plastic shell connecting to a 5.6 kw oil cooling system.

**FIGURE 2:**
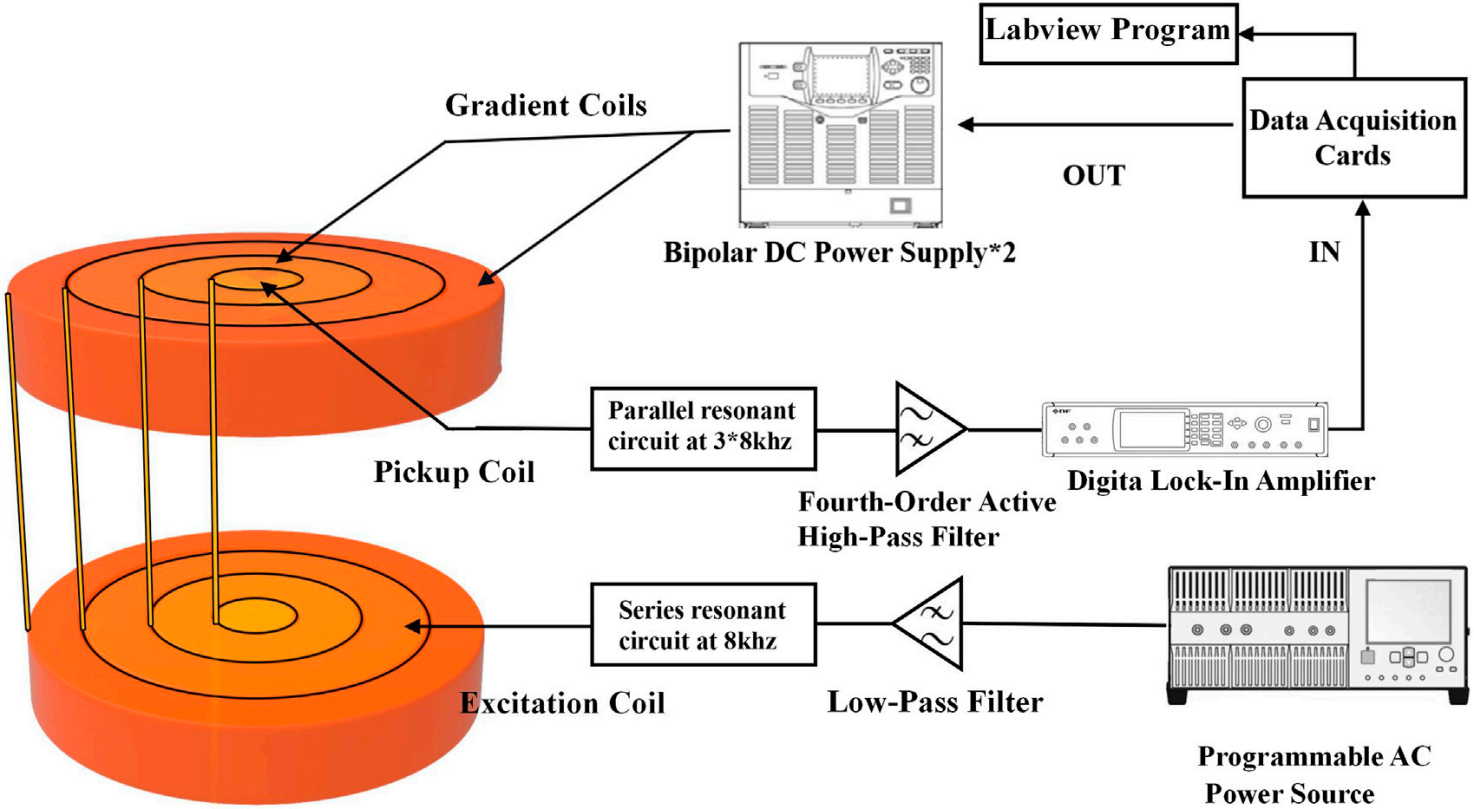
Diagram of the developed single-side SLNB MPI system. The differential coils were set far away from the original coils to avoid signal reduction caused by a too close baseline distance. Resonance circuits were connected to the excitation and pickup coils in serials and parallel in order to reduce the coil impedance and improve the detected third harmonic signal, respectively.

In this study, a high-resolution single-side MPI system was developed for SLNB application. Firstly, the gradient magnetic field consisted of two coaxial thin circular coils with average diameters of 160 mm and 270 mm, and coil turns of 600 and 508, respectively. Using different current combinations flowing in the inner and outer gradient coils, the FFP could be moved along the central axis with a uniform strength, as shown in Figure 1 and Table 1, which was very different from previous studies. Note that the directions of two gradient coils were opposite here. A gradient strength of about 0.54T/m was obtained from 0 to 30 mm depth in the Z direction, with a maximum 31.6A transient current, corresponding to a spatial resolution of about 5 mm in the narrow-band MPI that was discussed in previous studies. Considering the size of a lymph node is generally about 5 mm or more, the spatial resolution in the system developed is enough for SLNB diagnosis. Coils were packaged in a high strength plastic shell connecting to a 5.6 kw oil cooling system to lower the temperature at high currents. Secondly, the excitation coil was positioned between two gradient coils with a diameter of 210 mm. The flat-shaped excitation coil could generate a uniform excitation field with a maximum strength of 1.5 mT@8 kHz at 40 mm away from the coil surface, and the uniformity of the excitation field was more than 90% within the region bounded by a 40 mm radius around the central axis at each z distance. A series resonance circuit was used here to reduce the AC resistance of the excitation coil in 8 kHz. Finally, the pickup coil was placed inside the inner gradient coil with a diameter of 30 mm. A parallel resonance circuit was used to enhance the third harmonic signals from the SPIONs. What really counts here was that the differential pickup coil which was necessary in MPI detection was not suitable to set nearby the pickup coil because of the thickness of the excitation and gradient coils, which might cause significant signal degradation due to the too close base-line distance of the pickup and differential coils. Thus, in this study, a full mirrored coil set including all excitation, gradient, and pickup coils was set far away from the original one with a reverse series circuit in order to form a remote differential structure as shown in Figure 2. The third harmonic signal of magnetized SPIONs was detected using a lock-in amplifier, and then the *V*_3_ signal was reconstructed to Fe concentration using a home-made non-negative least square, NNLS program according to the 1D system matrix. The novel developed differential coils could suppress the system noise to about 100 dB without any loss of SPION signals. Another reason for placing the differential coils outside is the weight of the coils. At present, the anterior segment of the sensor carries a mass of approximately 40 kg, and the existing research robotic arm demonstrates insufficient load-bearing capacity. Consequently, we are actively engaged in outfitting the apparatus with an industrial-grade robotic arm, with the objective of expeditiously achieving comprehensive imaging of the scanning area.

**TABLE 1:** Movement of the FFP in the Z direction.

Spacing (mm)	Internal gradient coil current (A)	External gradient coil current (A)	Gradient magnetic field strength
0	15	14.8	0.55T/m
10	20	21.9	0.54T/m
20	25	24.8	0.54T/m
30	30	31.6	0.53T/m

### 2.3 *In vitro* experiment of developed system

**FIGURE 3:**
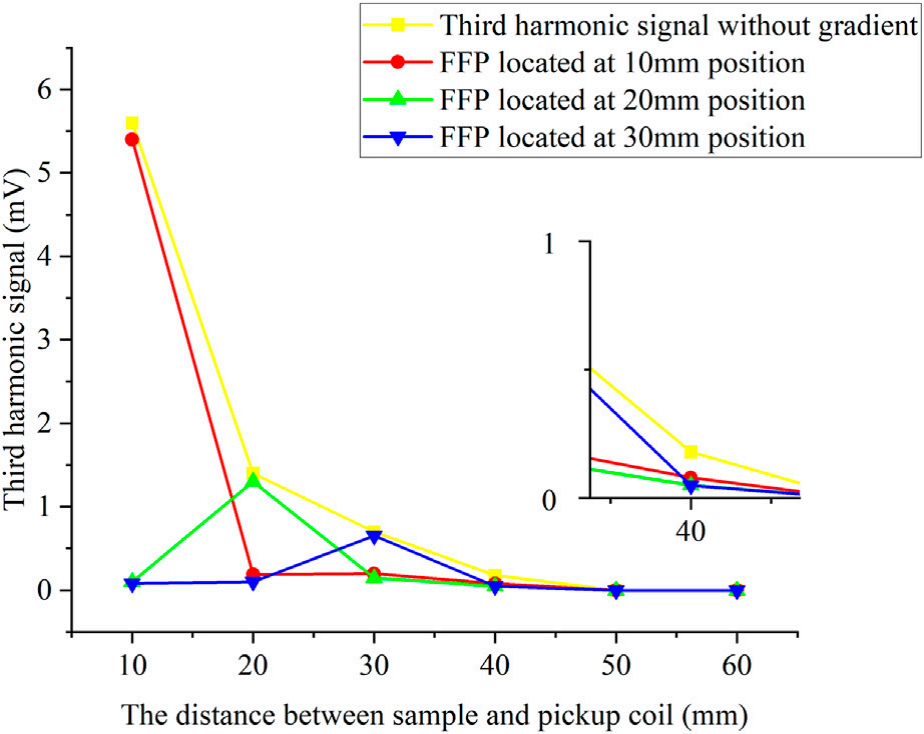
Third harmonic signals with and without gradient field. The amount of 10 μg Fe SPIONs sample in a Ø = 5 mm spherical container was set along the central axis of the coils with the Z distance from 10 to 60 mm, while the FFP was located at 10, 20, and 30 mm, respectively.

The amount of 10 μg Fe of suspended SPIONs sample in a *Ø* = 5 mm spherical container was set at the center line of the pickup coils with Z distance from 10 to 60 mm, while FFP was controlled to locate at 10 mm, 20 mm, and 30 mm, respectively. Figure 3 shows the measured *V*_3_ signal with and without gradient fields. Since the FFP is generated by the addition of a gradient magnetic field, the magnetic tracer generates a signal only at the FFP which is then detected by the pickup coil, and we controlled the movement of the FFP to facilitate tomography. As shown, the SPIONs sample was well detected in each FFP location, and the maximum detection distance was about 40 mm without gradient fields. The *V*_3_ results were also used as the standard 1D system matrix for Fe concentration reconstruction.

## 3 *In vivo* experiment of rats SLNB

Two types of experiments using rat animal models were conducted to evaluate the sensitivity and optimal detection time window of the developed MPI SLNB system.

**FIGURE 4:**
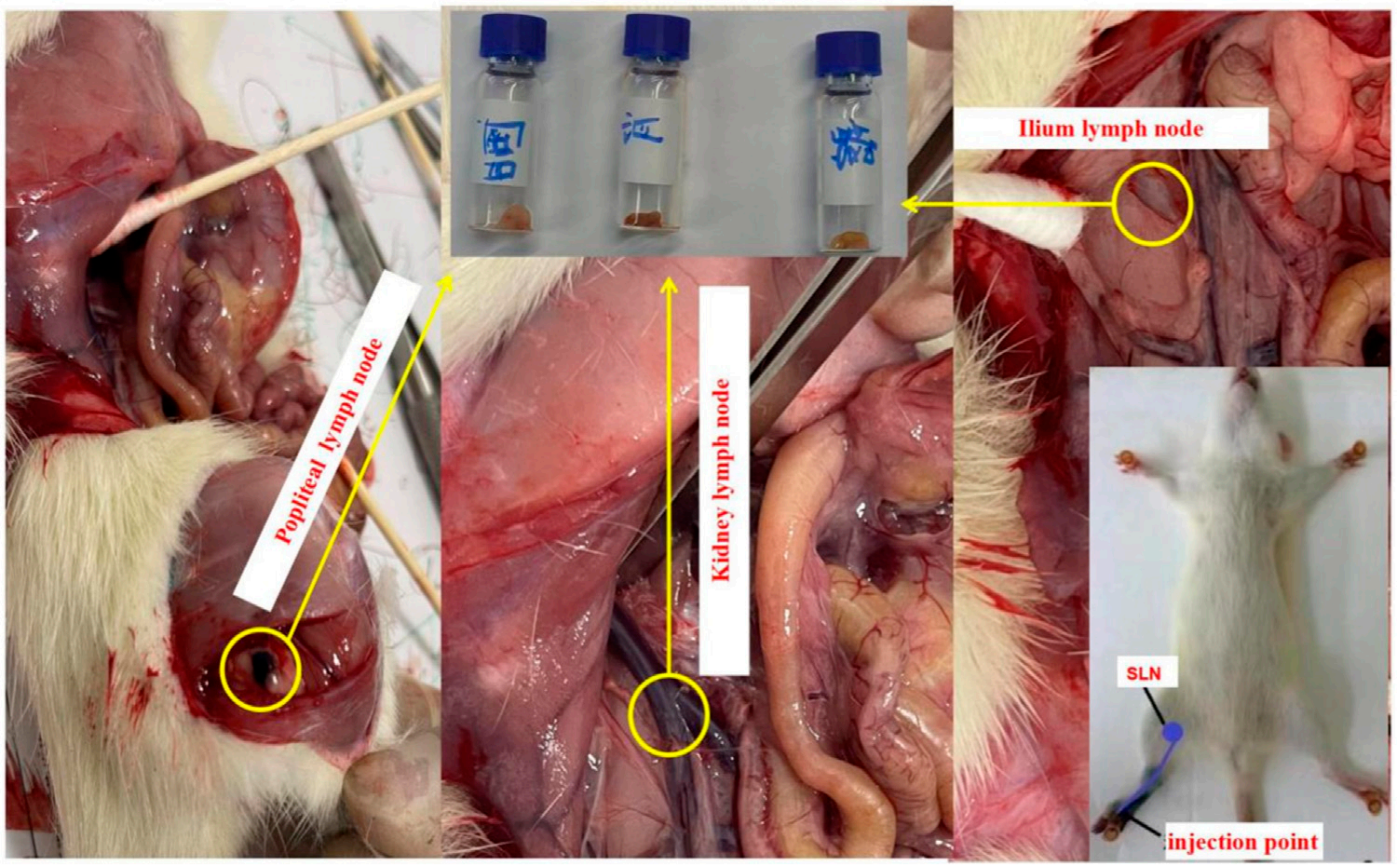
Tracer injection position and the sentinel and other lymph nodes of rats. All lymph nodes were taken out for further detection.

Experiment 1 The rat anesthesia procedure involved the addition of 1g of Pelltobarbitalum Natricum (Shanghai Chemical Reagent Co., Ltd., China) to a saline solution, resulting in a 100 mL preparation. The rats were then intraperitoneally injected with a dosage of 45 mg/kg. Once the rats exhibited unresponsiveness to stimuli, four healthy rats with an average weight of 500 g were subsequently injected with varying quantities of Resovist agent (Fujifilm, Japan). In the experiment, a volume of 0.05 mL of Resovist was diluted with 1 mL of physiological saline, resulting in a final concentration of 1.4 mg/mL Fe. Subsequently, injections of 0.12, 0.1, 0.08, and 0.04 mL of the solution were administered into the leg muscles of four rats. Following a 5 min massage, the popliteal fossa, ilium, kidney lymph node, and liver were excised and positioned at fixed distances (5, 10, 20, and 30 mm) from the developed MPI system. Figure 4 illustrates the injection sites and lymph node positions in the rat, with the popliteal fossa lymph node identified as the sentinel lymph node based on anatomical considerations.

Experiment 2 Following an anesthesia operation on rats, a group of five healthy rats with a mean weight of 500 g were administered identical doses of Resovist to determine the effective detection time. A 0.5 mL Resovist solution was diluted with 1 mL of physiological saline, and subsequently, 0.1 mL of the resulting solution was injected into the leg of each rat. Each rat was then massaged for a duration of 4–6 min, after which they were left undisturbed for intervals of 2, 4, 6, 10, and 30 min, respectively. The lymph nodes and liver were extracted and evaluated using the MPI system. The rats in Experiments 1, 2 were euthanized by injection of pentobarbital.

### 3.1 Ethical statement

All animals were kept in a pathogen-free environment and fed *ad lib*. The procedures for the care and use of animals were approved by the Ethics Committee of the Cancer Hospital of China Medical University and all applicable institutional and governmental regulations concerning the ethical use of animals were followed.

## 4 Results

### 4.1 Experimental results

**FIGURE 5:**
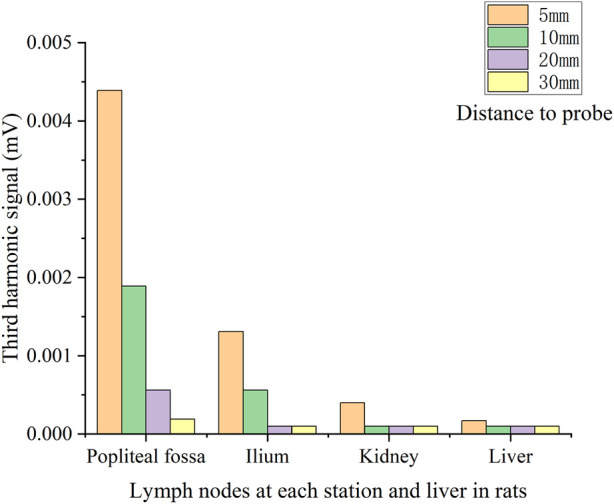
*V*_*3*_ signal of each lymph node located at different distances to the pickup coil of the first rats.

**FIGURE 6:**
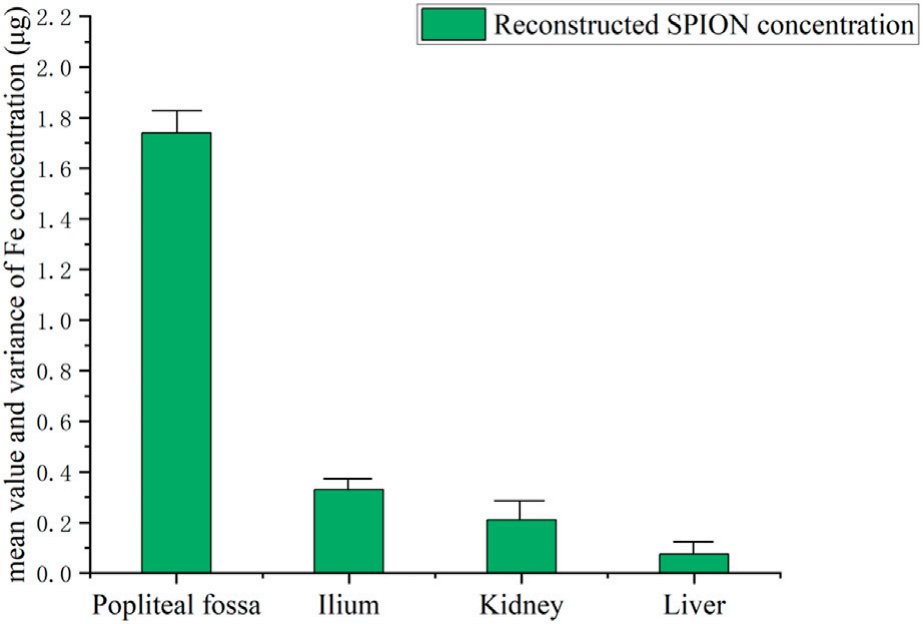
Reconstructed Fe concentration (mean and variance) in each lymph node.

Figure 4 shows the operation described in Experiment 1. Figure 5 illustrates the third harmonic signals of three lymph nodes and liver of the 0.12 mL case rat. Figure 6 gives the reconstructed Fe content. The reconstruction process was conducted at each scanning distance, and the estimated mean value and variance are displayed in Figure 6. The signals of Fe SPIONs were detected and used as a standard one-dimensional system matrix for reconstructing the iron concentration. The results demonstrated that all lymph nodes were detectable within a maximum distance of 30 mm, and the reconstructed value indicated that 1.74 μg Fe SPIONs (equivalent to approximately 1.2% of the injected content) had flowed into the SLN.

**FIGURE 7:**
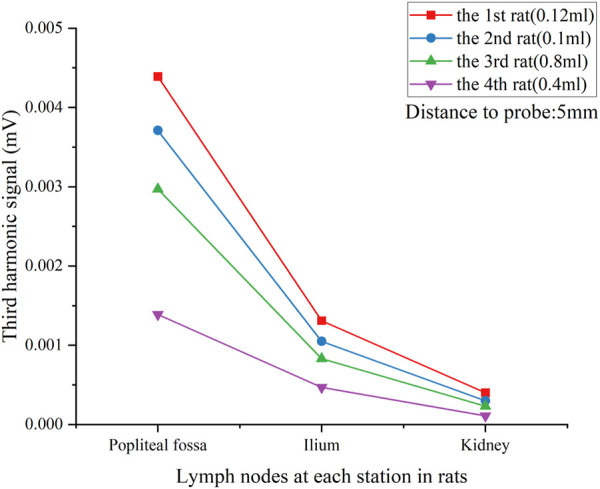
*V*_*3*_ signal of each lymph node in all rats with different tracer injection concentrations. Lymph nodes were located 5 mm from the pickup coil.

Figure 7 gives the comparison of the lymph nodes of four rats. SLN exhibited excellent observability in all case, indicating the minimum amount of intramuscularly injected Fe (54 μg or 0.04 mL) was sufficient for successful SLN biopsy identification. However, a larger injection quantity of SPIONs was much more suitable for SLNB diagnosis, as it resulted in a higher signal ratio between the SLN and system noise.

**FIGURE 8:**
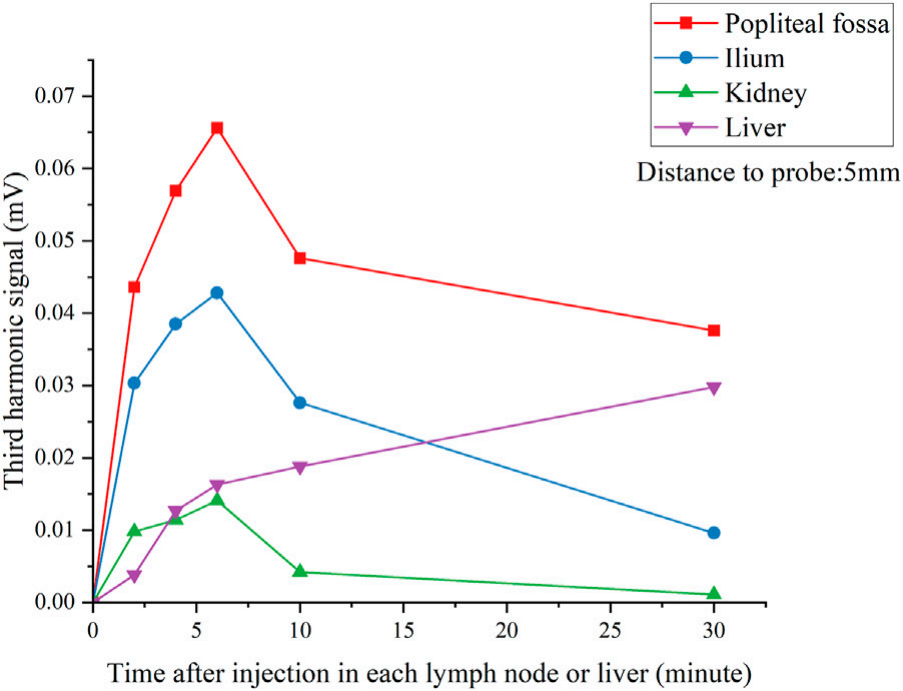
*V*_*3*_ signal of each lymph nodes and liver over time after injection. Lymph nodes were located 5 mm from the pickup coil.

Figure 8 shows the results of the effective detecting time window as mentioned in Experiment 2. We found that a maximum of the third harmonic signal was obtained between 4 and 6 min after injection for all lymph nodes, while the signal in the liver increased significantly in 30 min. Experimental results indicated that there was a time sensitivity to the SLNB MPI; this is consistent with the tracer’s metabolism through the liver in humans, which is particularly rapid in rats and SPIONs tracers could be metabolized well in the liver.

### 4.2 Discussion

Through the unique design of gradient fields and differential pick up system, the developed MPI system showed an expected sensitivity and tomographic ability in SLNB detection. The detection limit was about 10 μg Fe SPIONs in depths of 40 mm, and the minimum SLNB identification ability was 54 μg intramuscular injected Fe, according to a previous study; 1.6 mL intramuscular injection of Resovist in anterior lymph nodes contains about 140 ug Fe, so the sensitivity of this system can meet the clinical needs. In addition, since the safe dose of Resovist is 446 μg Fe/kg, the injection volume of 54 μg/500 g (rat body weight) in rats is much lower than the safe dose in humans. Comparing with the FDA approved magnetic SLNB device named Sentimag^®^, there is a comprehensive advantage in sensitivity, detection depth, and tomographic detection ability. Further, time sensitivity is another critical factor in MPI SLNB application, and the 4–6 min time window was like recent clinical studies.

However, 3D imaging (mechanical movement in the XY direction) was not carried out in the present study due to the small rat volume. The 0.54T/m field gradient of the system was set for human-sized SLNB but was not strong enough to provide the spatial resolution for small animal imaging. Large mammalian SLNB MPI experiments and 3D preoperative SLNB imaging will be performed in the future.

## 5 Conclusion

In this study, a human-size SLNB MPI system was developed. Experimental results using rats indicated that the developed system showed excellent ability for SLNB identification. Large-scale animal and human clinical trials based on the developed system are in preparation. We expect this to be the first MPI clinical solution for SLNB application in future.

## Data Availability

The raw data supporting the conclusion of this article will be made available by the authors, without undue reservation.
